# Correcting for day of the week and public holiday effects: improving a national daily syndromic surveillance service for detecting public health threats

**DOI:** 10.1186/s12889-017-4372-y

**Published:** 2017-05-19

**Authors:** Elizabeth Buckingham-Jeffery, Roger Morbey, Thomas House, Alex J. Elliot, Sally Harcourt, Gillian E. Smith

**Affiliations:** 1 Centre for Complexity Science and Warwick Infectious Disease Epidemiology Research Centre, University of Warwick, Coventry, UK; 2 Real-time Syndromic Surveillance Team, National Infection Service, Public Health England, Birmingham, UK; 3 School of Mathematics, University of Manchester, Manchester, UK

**Keywords:** Syndromic surveillance, Day-of-the-week effect, Smoothing

## Abstract

**Background:**

As service provision and patient behaviour varies by day, healthcare data used for public health surveillance can exhibit large day of the week effects. These regular effects are further complicated by the impact of public holidays. Real-time syndromic surveillance requires the daily analysis of a range of healthcare data sources, including family doctor consultations (called general practitioners, or GPs, in the UK). Failure to adjust for such reporting biases during analysis of syndromic GP surveillance data could lead to misinterpretations including false alarms or delays in the detection of outbreaks.

The simplest smoothing method to remove a day of the week effect from daily time series data is a 7-day moving average. Public Health England developed the working day moving average in an attempt also to remove public holiday effects from daily GP data. However, neither of these methods adequately account for the combination of day of the week and public holiday effects.

**Methods:**

The extended working day moving average was developed. This is a further data-driven method for adding a smooth trend curve to a time series graph of daily healthcare data, that aims to take both public holiday and day of the week effects into account. It is based on the assumption that the number of people seeking healthcare services is a combination of illness levels/severity and the ability or desire of patients to seek healthcare each day. The extended working day moving average was compared to the seven-day and working day moving averages through application to data from two syndromic indicators from the GP in-hours syndromic surveillance system managed by Public Health England.

**Results:**

The extended working day moving average successfully smoothed the syndromic healthcare data by taking into account the combined day of the week and public holiday effects. In comparison, the seven-day and working day moving averages were unable to account for all these effects, which led to misleading smoothing curves.

**Conclusions:**

The results from this study make it possible to identify trends and unusual activity in syndromic surveillance data from GP services in real-time independently of the effects caused by day of the week and public holidays, thereby improving the public health action resulting from the analysis of these data.

## Background

Syndromic surveillance is the near real-time collection, analysis, interpretation, and dissemination of health related data to enable the early identification of the impact of potential public health threats [1]. The real-time syndromic surveillance team at Public Health England (PHE) co-ordinates a suite of syndromic surveillance systems in order to provide early warning of outbreaks of infectious disease, situational awareness during a public health incident, and reassurance of lack of impact [2–5]. These syndromic surveillance systems are used to complement and support existing public health surveillance programmes.

Line graphs of time series data offer a simple and effective way to review data and undertake exploratory analysis [6, 7]. They are used, in addition to automated statistical alarms, by the real-time syndromic surveillance team to investigate, interpret, and present the current trends in syndromic data and for comparisons of the current data with previous years to identify changes from the norm. Regular, large fluctuations at small time-scales can, however, make it difficult to identify longer time-period trends in time series graphs. These difficulties can be overcome by adding to the graph a smooth trend curve which takes into account these known day-to-day fluctuations [8].

The *GP in-hours syndromic surveillance system* (GP in-hours SSS) monitors the number of in-hours family doctor (known as general practitioner, or GP, in the UK) consultations [9]. Daily data on the number of GP consultations are analysed, and are aggregated into *syndromic indicators* based on symptoms and clinical diagnoses (e.g. influenza-like illness, diarrhoea, chickenpox) [9]. Although much of the GP in-hours SSS is automated, statistical alarms are created that require manual, in-depth investigation [10]. Effective data visualisations must be used in order for the manual investigation stage not to become the bottleneck of the real-time data analysis process [11].

Graphs of the syndromic indicators from the GP in-hours SSS are presented to the public and wider audiences in weekly bulletins published by PHE [12]. This is an additional reason to ensure that the current trend in illness levels can be clearly interpreted from the graph without additional data or expert knowledge.

Regular fluctuations at a weekly time-scale, known as *day of the week effects*, have been observed in the number of patient consultations with GP services [10]. The number of consultations is also observed to regularly change on a public holiday and on the days immediately after [10]. We refer to this as a *public holiday effect.*

The purpose of syndromic surveillance is to identify abnormally elevated disease levels as early as possible so that action can be taken to minimise the problem [13, 14]. However, if the systematic changes in the number of consultations with GPs due to day of the week and public holidays are not accounted for, they could mask real increases in disease levels, create false alarms, and delay decision making over public holiday periods as more data are required to understand the current trend. It is important to try to distinguish the expected changes in consultation numbers due to day of the week or public holiday effects from unexpected changes due to potential public health threats.

The purpose of this work is to develop and explore an appropriate smoothing method that takes the expected day of the week and public holiday effects into account simultaneously and displays no trend due to these predictable variations. This method will be applied to time series graphs to enhance visual analysis of daily GP consultation data for syndromic surveillance. This will improve daily risk assessments by epidemiological investigators.

Data from healthcare services reflect the time at which patients sought healthcare advice. This does not necessarily correspond with date of symptom onset. In particular, patients with milder illnesses may not present unless they become more severe or complications develop [15, 16]. Therefore, the number of healthcare consultations is not a simple measure of illness in the population but rather a combination of illness levels, severity of the illness, availability of healthcare services, and ability or willingness to seek healthcare [17]. Based on this, we develop a data-driven smoothing method, the *extended working day moving average,* using scaling factors to take both day of the week and public holiday effects into account.

The rest of this paper is organised as follows. The Background will conclude with a short discussion of the existing literature of smoothing methods to account for day of the week and public holiday effects in healthcare data, a description of the specific calendar effects observed in the GP in-hours SSS, and a description of the seven-day and working day moving average. The limitations of these methods justify the development of the extended working day moving average to take day of the week and public holiday effects into account simultaneously, which will be described in the Methods section. This will be followed by a description of the data from the GP in-hours SSS to which the smoothing methods will be applied. An evaluation of the extended working day moving average, with comparison to the seven-day and working day moving averages will be presented in the Results section. Finally, the strengths and limitations of the smoothing methods and the impact of using the extended working day moving average on public health practice will be discussed.

### Existing literature of smoothing methods to account for day of the week and public holiday effects in healthcare data

Smoothing to remove day of the week effects and visualise trends has been noted as being important for analysis of healthcare data [18–22], although few smoothing methodologies have specifically been developed to enhance visual interpretations in this context. However, both model-based and data-driven smoothing methods have been used to remove day of the week and/or public holiday effects as part of more complex detection algorithms [17].

Many published methodologies are able to smooth day of the week effects but do not consider public holiday effects [17, 22, 23]. However, this study will demonstrate that both day of the week and public holiday effects must be considered simultaneously to enable continued, effective surveillance of GP consultation data during and around public holidays.

The working day moving average was developed by PHE to visualise trends in syndromic data from the GP in-hours SSS, however this has not previously been described in the literature.

### Day of the week and public holiday effects in the GP in-hours SSS

**Fig. 1:**
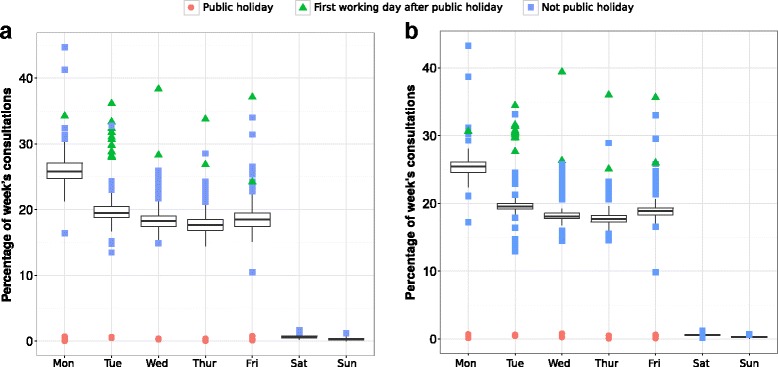
Box plots of data from the GP in-hours syndromic surveillance system demonstrating the day of the week and public holiday effects for the (**a**) severe asthma indicator and (**b**) gastroenteritis indicator. Daily consultation numbers for each day between 2nd April 2012 and 11th January 2015 were grouped into weeks from Monday to Sunday and the proportion of the week's consultations on each day of the week are summarised in the box plots

In the GP in-hours SSS more consultations occur on Monday than on any other day of the week. There were typically fewer consultations on each of Tuesday through Friday, and a negligible number of consultations on weekends. Figure 1 displays, as examples, the proportion of the week’s consultations (Monday – Sunday) on each day of the week, for the severe asthma and gastroenteritis indicators. On all public holidays there were a negligible number of consultations (Fig. 1), and the first working day after a public holiday typically had a higher number of consultations than expected for the day of the week.

### Description of smoothing methods used for comparisons

A 7-day moving average is the simplest data-driven smoothing approach to remove a day of the week effect. No adjustment is made for public holiday effects in this method.

A moving average is a series of averages of subsets of the time series of syndromic data. The first element of a 7-day moving average is the average of the first seven data points. The second element is the average of the second to eighth data point. This is continued so that each set of seven consecutive data points is averaged [24]. Seven days was chosen in this context as day of the week effects have 7-day periodicity.

The working day moving average method was previously developed by PHE to take both day of the week and public holiday effects into account when visualising data from syndromic surveillance systems. This simple adjustment of the 7-day moving average aims to take into account public holidays and ensure the smoothing line takes values similar to the number of consultations on an average working day.

The working day moving average is constructed as follows. Due to reduced opening hours, very few routine in-hours GP consultations occur on public holidays. Therefore, public holidays are grouped with weekends, and a moving average is computed that takes into account the number of working days. Let *n* denote the number of working days within the current block of 7 days being considered to give an element of the moving average. In the GP in-hours SSS this is typically five, as doctors’ surgeries do not typically open on weekends. However, in blocks containing public holidays it will be fewer. Instead of simply computing the average of the number of consultations on the 7 days, the sum of the number of consultations on working days was multiplied by $\frac{5}{n}$ and the sum of the number of consultations on non-working days was multiplied by $\frac{2}{7- n}$. The sum of these totals was then divided by five, the typical number of working days in the GP in-hours SSS.

For a block of 7 days with no public holidays, this calculation just gives $\frac{1}{5}$ times the sum of the number of consultations on the 7 days in question, a basic moving average. For blocks of 7 days containing public holidays, this calculation weights the working days slightly more than the simple sum and the non-working days slightly less. This accounts for the expected reduction in total consultations in the week due to the public holiday.

## Methods

### Extended working day moving average

In the extended working day moving average, we do not simply assume that healthcare seeking behaviour on public holidays is the same as on weekend days and that behaviour on all other weekdays is the same. Instead, each different day of the week and each day affected by a public holiday is assigned a scaling factor. This simultaneously takes into account changes in the number of healthcare consultations on days surrounding public holidays, changes in the number of consultations on the public holiday itself, and the day of the week effect.

Data from one complete year, excluding any weeks containing public holidays, were used to give the scaling factors of the extended working day moving average for a syndromic indicator from the GP in-hours SSS. Therefore, the scaling factors will be different for each syndromic indicator.

In order to compute the scaling factors, the proportion of each week’s activity (Monday – Sunday) on each day was calculated. These were averaged over all weeks not containing public holidays to give an average proportion of the weekly activity on each day of the week. These average proportions were multiplied by five, the number of working days in a typical week in the GP in-hours SSS, to give the initial scaling factors. Additional scaling factors were developed based on the public holiday effects. Each public holiday was assigned the same scaling factor as a typical Sunday, and the first working day after a public holiday was given the same scaling factor as a typical Monday. These scaling factors reflect the typical number of consultations on each day of the week; a value larger than one reflects a day with typically a higher than average number of consultations.

To construct the extended working day moving average, the sum of each 7-day block was divided by the sum of the corresponding scaling factors. Note that the extended working day moving average for a 7-day block without a public holiday is simply the sum of consultations divided by five, giving a basic moving average during these periods.

### Data

The extended working day moving average has been developed for smoothing data from the GP in-hours SSS. However, the dynamics of the diseases that generate the syndromic data are complex, and the recorded activity levels are affected by system coverage fluctuations, data collection changes, and other unknown influences on top of the day of the week and public holidays effects [10]. This can make it difficult to clearly compare and evaluate the different smoothing methods. Therefore, they were first applied to synthetic data with the same public holiday and day of the week effects as the GP in-hours SSS but without longer-term trends and noise.

**Fig. 2:**
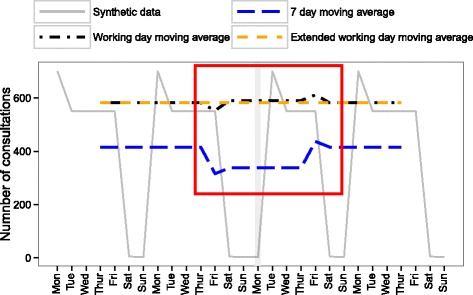
The extended working day moving average applied to synthetic data, with the seven-day and working day moving averages for comparison. Synthetic data were generated for 28 days, containing day of the week and public holiday effects representative of those observed in the GP in-hours syndromic surveillance system, but without noise and longer term trends. The synthetic data included a public holiday Monday. This is indicated by the grey vertical line and easily identifiable by the negligible number of consultations on this day. The extended working day moving average was applied to this data with the seven-day and working day moving average shown for comparison. The *red box* highlights the pre- and post- public holiday period of interest

We constructed synthetic data for a period of 4 weeks. Based on historic data, we considered a total of 2900 consultations per week and split this into 696 consultations on Monday (24% of the week’s consultations), 522 (18%) on each of Tuesday to Friday, and 58 (2%) on weekend days. In order to incorporate a public holiday effect, the third Monday of the synthetic data was denoted as a public holiday. This day was given the same number of consultations as a Sunday (58 consultations, or 2.4% of the public holiday week’s consultations). The Tuesday immediately after was given the same number of consultations as the typical Mondays (696 consultations, or 28.6%). The number of consultations on all other days in this week was left unchanged (522, or 21.4%, on the remaining weekdays and 52, or 2.4%, on the weekend days). There were fewer consultations overall in the week containing the public holiday. The synthetic data are presented in Fig. 2.

The smoothing methods were also applied to actual data from the GP in-hours SSS for 52 weeks, from 13th January 2014 to 11th January 2015. The indicators severe asthma and gastroenteritis were chosen as examples. Other syndromic indicators could have been used; similar day of the week and public holiday effects are extensively observed across the system.

## Results

As previously described, the extended working day moving average was applied to synthetic data and the severe asthma and gastroenteritis syndromic indicators from the GP in-hours SSS. The 7-day and working day moving averages were also applied for comparison.

Using the percentages 2%, 18%, and 24% described in the Data section, the scaling factors for the extended working day moving average applied to the synthetic data were calculated as 0.1 for weekends and public holidays, 1.2 for typical Mondays and the first working day after a public holiday, and 0.9 for all other typical weekdays. The scaling factors calculated from the severe asthma and gastroenteritis indicator data are given in Table 1.

**Table 1:** Scaling factors for indicators from the GP in-hours syndromic surveillance system for the extended working day moving average

	Scaling factors: severe asthma	Scaling factors: gastroenteritis
Monday	1.30	1.25
Tuesday	0.95	0.95
Wednesday	0.91	0.91
Thursday	0.87	0.90
Friday	0.93	0.95
Saturday	0.03	0.02
Sunday	0.01	0.01
Public holiday	0.01	0.01
First working day after public holiday	1.30	1.25

The scaling factors for the extended working day moving average for Monday – Sunday were based on 52 weeks of data (13th January 2014 - 11th January 2015) using the method outlined in the main text. The scaling factors for public holidays and their surrounding days were based on observations made of the GP in-hours syndromic surveillance system over multiple years

**Fig. 3:**
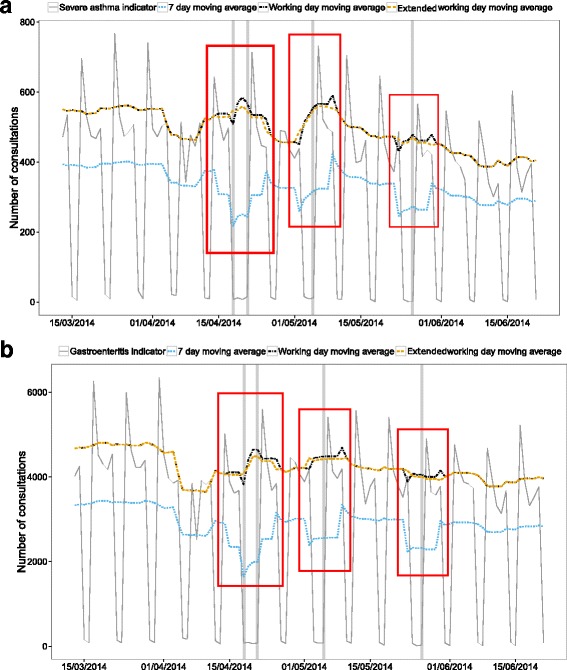
The number of (**a**) severe asthma and (**b**) gastroenteritis consultations from the GP in-hours syndromic surveillance system with the extended working day moving average. The seven-day and working day moving averages are also included for comparison. The grey vertical lines indicate public holidays. The *red boxes* highlight the pre- and post- Monday public holiday dips and peaks in the seven-day and working day moving average and their removal in the extended working day moving average

The extended working day moving average showed a no-trend line when applied to the synthetic data, as the combination of day of the week and public holiday effects were taken into account (Fig. 2). The extended working day moving average also continued to display the trends in the syndromic data throughout public holiday periods (Fig. 3).

In the absence of public holidays, the seven-day moving average applied to the synthetic data smoothed the regular day of the week effect to highlight the current trend. However, there is a dip in the smoothing trend curve for 7 days around the public holiday (Fig. 2). These synthetic data followed the expected behaviour of no-trend syndromic data around a public holiday. With real data, this dip in the smoothing curve could mask an actual increase in disease levels over this time period. However, this change is entirely expected due to the change in healthcare service provision on public holidays. Additionally, the 7-day moving average was lower than the average number of consultations on a working day. It is more useful that the smooth trend curve gives an indication of the number of healthcare contacts on a typical working day.

These same results were also observed when the 7-day moving average was applied to surveillance data for the severe asthma and gastroenteritis indicators (Fig. 3).

The working day moving average applied to synthetic data gave a better smooth curve than the 7-day moving average (Fig. 2). However, a drop 3 days before and a peak 4 days after public holidays were still present in the smoothing curve when applied to both synthetic and real data (Figs. 2 and 3). These were due to the combination of the day of the week and public holiday effects. The drop was caused by that 7-day sum not including a typical Monday, and the peak was caused by that 7-day sum including both a typical Monday and the elevated Tuesday directly after the public holiday.

In the absence of big day of the week effects, the working day moving average would smooth a simple public holiday effect. However, the interaction between day of the week and public holiday effects, and extended holiday effects such as a change in activity on the first working day after a public holiday, are not accounted for.

**Fig. 4:**
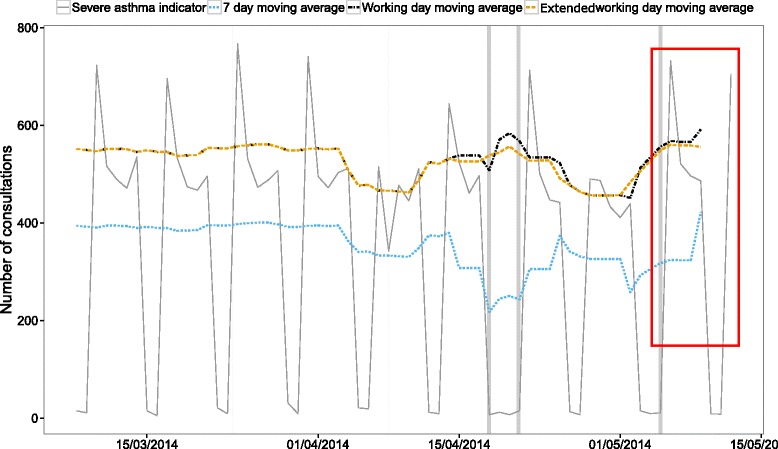
A comparison of the current trend given by each of the smoothing methods for the severe asthma indicator from the GP in-hours syndromic surveillance system. This graph displays the data that is available 1 week after a Monday public holiday (public holidays indicated by *grey vertical lines*). A smoothing method would be used to display the current trend (the area of interest inside the *red box*). Both the seven-day and working day moving averages show a currently increasing trend. The extended working day moving average and, importantly, the data do not

Smoothing trend curves are used to help investigators visually identify current unusual activity during daily surveillance of syndromic disease data. It is easy to retrospectively look at the smoothing curve given by the working day moving average and identify the spikes as clearly spurious due to their short duration. However, in order to emphasise how misleading the 7-day and working day moving averages can be we applied all the smoothing methods to the dataset that would be available a week after a Monday public holiday. This graph would be used to assess the current trend in the number of severe asthma consultations (Fig. 4). The trend 1 week after a public holiday would be noted as increasing if either the 7-day or working day moving averages were used. This could lead to unnecessary alarm. The extended working day moving average did not show an increasing trend and, more importantly, neither did the data. The extended working day moving average would make it easier for investigators to identify unusual activity during this period.

## Discussion

It is widely acknowledged that day of the week and public holiday effects exist in healthcare data used for syndromic surveillance and that this can disguise anomalies in the data when visually inspecting it [10, 17–23]. In this study, we described the previous smoothing method used by PHE to smooth data from the GP in-hours SSS. We also developed a smoothing method where both day of the week and public holiday effects are taken into account simultaneously. We demonstrated how the extended working day moving average can be used to aid interpretation of the trends in real-time syndromic surveillance data from GP services, thereby improving the public health action resulting from the analysis. The extended working day moving average method retains the ability to display unusual changes in the trends of syndromic indicators from the GP in-hours SSS during public holiday periods, and it removes the potentially misleading spikes observed in the working day moving average. This reduces the potential for delays in the detection of public health threats during this time.

The inter-quartile ranges of the proportion of consultations on each day of the week are quite narrow (Fig. 1). This indicates that the day of the week effect is consistent throughout the year. However, day of the week and public holiday effects are just one cause of noise in these complex data sets. The number of GP consultations fluctuates and contains regular trends due to other factors that we do not discuss or control for here. These include, for example, seasonal disease outbreaks and changes in the data collection systems.

In this study only relatively simple data-driven smoothing methods were considered. Syndromic surveillance uses large, varied data sets, and it is desirable for syndromic surveillance reporting systems to be as automated as possible. A simple data-driven smoothing approach ensures sufficient flexibility so that smoothing methods can be applied to a wide range of indicators in an automated way [25]. As discussed in the Background, data-driven smoothing methods have previously been used to remove day of the week and/or public holiday effects from daily syndromic data as part of more complex detection algorithms [17, 20, 26, 27]. However, this study shows that both day of the week and public holiday effects must be considered simultaneously to create adequately smooth daily healthcare data. We have addressed this problem in the context of GP in-hours consultation data used for daily syndromic surveillance in England, and we have focused on methods to improve time series graphs used for daily risk assessments by investigators.

The extended working day moving average was developed for the GP in-hours SSS coordinated by PHE. We demonstrated the method applied to the gastroenteritis and severe asthma indicators as examples. However, the day of the week and public holiday effects observed in these two indicators are also observed across the GP in-hours SSS in a consistent way (see, for example, the plots of data for a large number of indicators within the PHE weekly bulletin [12]). It is therefore appropriate and straightforward to apply the method to other syndromic indicators from the GP in-hours SSS, and we see the same results as discussed here. As a result of this, the extended working day moving average is now in use across the GP in-hours SSS.

Day of the week or public holiday effects are also seen in attendance data from many other healthcare services. This includes emergency departments [28], walk-in clinics [29], military treatment facilities [15], sexual health clinics [30], telehealth services [5], and internet based symptom-checker services [31]. It is also seen in the other syndromic surveillance systems operated by PHE. This work has demonstrated the importance of being aware of day of the week and public holiday effects in analysis and interpretation of this type of data, including the effect on days near to the public holiday itself. We have shown how an inadequate treatment of these effects can lead to potential confusion in the current trend and delay decision making.

However, the extended working day moving average described here was developed for use with just one particular syndromic surveillance system. Further work is needed to investigate whether the extended working day moving average could be applied to other surveillance systems. In particular, whether it is valid for those which monitor attendances at 7-day healthcare services. Additionally, if the day of the week and public holiday effects are not as large as those observed in the GP in-hours SSS a simpler method could be sufficient. Further work in this area will describe the extent of the day of the week and public holiday effects across different syndromic surveillance systems. This will also involve an investigation of the public health aspects of these effects, rather than purely the statistical approaches considered during this analysis.

The main limitation of the extended working day moving average is that historical data are needed to compute the scaling factors. In particular, sufficient data are required to learn how the number of consultations changes around each public holiday. On the other hand, the working day moving average and 7-day moving average do not require historical data and therefore can be used immediately with new syndromic surveillance systems.

## Conclusions

Our results show that basic smoothing techniques are not able to account fully for the public holiday effects observed in the GP in-hours SSS. We have developed and demonstrated an improved smoothing technique that can make it easier for investigators to identify unusual activity during daily surveillance of syndromic GP data. This method is now in use in the GP in-hours SSS at PHE. It has led to enhanced visualisations of this data during the analysis phase and in weekly public health bulletins [12].

Based on this study, it is recommended that analysis and visualisation methods for syndromic data carefully take both day of the week and public holiday effects into account.

## Abbreviations

GP: General practitioner
PHE: Public Health England
SSS: Syndromic surveillance system

## References

[1] Triple S Project (2011). Assessment of syndromic surveillance in Europe. Lancet.

[2] Elliot AJ, Morbey RA, Hughes HE, Harcourt SE, Smith S, Loveridge P, Edeghere O, Ibbotson S, McCloskey B, Catchpole M (2013). Syndromic surveillance - a public health legacy of the London 2012 Olympic and Paralympic Games. Public Health.

[3] Harcourt SE, Fletcher J, Loveridge P, Bains A, Morbey R, Yeates A, McCloskey B, Smyth B, Ibbotson S, Smith GE (2012). Developing a new syndromic surveillance system for the London 2012 Olympic and Paralympic Games. Epidemiol Infect.

[4] Elliot AJ, Hughes HE, Hughes TC, Locker TE, Shannon T, Heyworth J, Wapling A, Catchpole M, Ibbotson S, McCloskey B (2012). Establishing an emergency department syndromic surveillance system to support the London 2012 Olympic and Paralympic Games. Emerg Med J.

[5] Harcourt SE, Morbey RA, Loveridge P, Carrilho L, Baynham D, Povey E, Fox P, Rutter J, Moores P, Tiffen J, et al. Developing and validating a new national remote health advice syndromic surveillance system in England. J Public health. 2016;39(1):184–92. 10.1093/pubmed/fdw013 PMC6092922 26956114

[6] Muller W, Schumann H (2003). Visualization for modeling and simulation: visualization methods for time-dependent data - an overview. Proceedings of the 35th conference on Winter simulation: driving innovation.

[7] Hauenstein L, Wojcik R, Loschen W, Ashar R, Sniegoski C, Tabernero N. Putting it together: the biosurveillance information system. In: Lombardo JS, Buckeridge DL, editors. Disease Surveillance A Public Health Informatics Approach. NJ: John Wiley & Sons Inc; 2007.

[8] Erbas B, Hyndman R (2001). Data visualisation for time series in environmental epidemiology. Journal of Epidemiology and Biostats.

[9] Harcourt SE, Smith GE, Elliot AJ, Pebody R, Charlett A, Ibbotson S, Regan M, Hippisley-Cox J (2012). Use of a large general practice syndromic surveillance system to monitor the progress of the influenza A(H1N1) pandemic 2009 in the UK. Epidemiol Infect.

[10] Morbey RA, Elliot AJ, Charlett A, Verlander NQ, Andrews N, Smith GE. The application of a novel ‘rising activity, multi-level mixed effects, indicator emphasis’ (RAMMIE) method for syndromic surveillance in England. Bioinformatics. 2015;31(22):3660–5. 10.1093/bioinformatics/btv418 26198105

[11] Moore KM, Edge G, Kurc AR (2008). Visualization techniques and graphical user interfaces in syndromic surveillance systems. Summary from the Disease Surveillance Workshop, Sept. 11–12, 2007; Bangkok, Thailand. BMC Proc.

[12] Research and Analysis: GP in hours bulletin. https://www.gov.uk/government/publications/gp-in-hours-bulletin. Accessed 12 May 2017.

[13] Mandl KD, Overhage JM, Wagner MM, Lober WB, Sebastiani P, Mostashari F, Pavlin JA, Gesteland PH, Treadwell T, Koski E (2004). Implementing Syndromic Surveillance: A Practical Guide Informed by the Early Experience. J Am Med Inform Assoc.

[14] Chretien JP, Burkom HS, Sedyaningsih ER, Larasati RP, Lescano AG, Mundaca CC, Blazes DL, Munayco CV, Coberly JS, Ashar RJ (2008). Syndromic surveillance: adapting innovations to developing settings. PLoS Med.

[15] Riley P, Cost AA, Riley S (2016). Intra-Weekly Variations of Influenza-Like Illness in Military Populations. Mil Med.

[16] Fleming DM, Elliot AJ (2008). Lessons from 40 years’ surveillance of influenza in England and Wales. Epidemiology & Infection.

[17] Wong W-K, Moore AW. Classical Time-Series Methods for Biosurveillance. In: Wagner MM, Moore AW, Aryel RM, editors. Handbook of Biosurveillance. MA: Elsevier Academic Press; 2006.

[18] Bollaerts K, Antoine J, Robesyn E, Van Proeyen L, Vomberg J, Feys E, De Decker E, Catry B (2010). Timeliness of syndromic influenza surveillance through work and school absenteeism. Archives of Public Health.

[19] Burkom HS, Murphy SP, Shmueli G (2007). Automated time series forecasting for biosurveillance. Stat Med.

[20] Forsberg L, Jeffery C, Ozonoff A, Pagano M, Wilson A, Wilson G, Olwell D (2006). A Spatiotemporal Analysis of Syndromic Data for Biosurveillance. Statistical Methods in Counterterrorism.

[21] Shmueli G, Burkom HS (2006). Statistical challenges in modern biosurveillance. Technometrics.

[22] Wijk JJV, Selow ERV. Cluster and calendar based visualization of time series data. In: Information Visualization, 1999 (Info Vis ‘99) Proceedings 1999 IEEE Symposium; 1999. p. 4–9, 140.

[23] Maciejewski R, Rudolph S, Grannis SJ, Ebert DS. The day-of-the-week effect: a study across the Indiana Public Health Emergency Surveillance System. International Society for Disease Surveillance Annual Conference Advances in Disease Surveillance 2008, 5(44).

[24] Engineering Statistics Handbook: e-Handbook of Statistical Methods. http://www.itl.nist.gov/div898/handbook/. Accessed 12 May 2017.

[25] Shmueli G, Burkom H (2010). Statistical Challenges Facing Early Outbreak Detection in Biosurveillance. Technometrics.

[26] Lotze T, Shmueli G, Murphy S, Burkom H. A wavelet-based anomaly detector for early detection of disease outbreaks. In: Workshop on Machine Learning Algorithms for Surveillance and Event Detection, 23rd Intl Conference on Machine Learning; 2006.

[27] Siegrist D, McClellan G, Campbell M, Foster V, Burkom H, Hogan W, Cheng K, Buckeridge D, Pavlin J, Kress A. Evaluation of algorithms for outbreak detection using clinical data from five us cities. VA: Technical report, DARPA Bio-ALIRT Program; 2004.

[28] Batal H, Tench J, McMillan S, Adams J, Mehler PS (2001). Predicting Patient Visits to an Urgent Care Clinic Using Calendar Variables. Acad Emerg Med.

[29] Holleman DR, Bowling RL, Gathy C (1996). Predicting daily visits to a waik-in clinic and emergency department using calendar and weather data. J Gen Intern Med.

[30] Gamagedara N, Hocking JS, Law M, Fehler G, Chen MY, Bradshaw CS, Fairley CK (2014). What are seasonal and meteorological factors are associated with the number of attendees at a sexual health service? An observational study between 2002–2012. Sex Transm Infect.

[31] Elliot AJ, Kara EO, Loveridge P, Bawa Z, Morbey RA, Moth M, Large S, Smith GE (2015). Internet-based remote health self-checker symptom data as an adjuvant to a national syndromic surveillance system. Epidemiology & Infection.

